# Science Revealing Ancient Magic: Phytolith Evidence from the Early Chalcolithic Site of Isaiia (Eastern Romania)

**DOI:** 10.3390/biology11081102

**Published:** 2022-07-23

**Authors:** Felix Adrian Tencariu, Claire Delhon, Diana Măriuca Vornicu, Andrei Asăndulesei, Casandra Brașoveanu, Mihaela Danu

**Affiliations:** 1 Arheoinvest Centre, Department of Exact and Natural Sciences, Institute of Interdisciplinary Research, Alexandru Ioan Cuza University of Iasi, 700506 Iasi, Romania; aditen@uaic.ro (F.A.T.); andrei.asandulesei@uaic.ro (A.A.); casandra.brasoveanu@uaic.ro (C.B.); 2 Centre National de la Recherche Scientifique, Cultures Environnements Préhistoire-Antiquité-Moyen Âge, Université Côte d’Azur, 06300 Nice, France; claire.delhon@cepam.cnrs.fr; 3 Institute of Archaeology, Romanian Academy-Iasi Branch, 700479 Iasi, Romania; mariucav@gmail.com; 4 Faculty of Biology, Alexandru Ioan Cuza University of Iasi, 700506 Iasi, Romania

**Keywords:** phytoliths, Isaiia–*Balta Popii* site, Romania, Early Chalcolithic, ritual deposition, magical plants

## Abstract

**Simple Summary:**

The present paper aims to present the newsworthy results and interpretation of the interdisciplinary analysis conducted at an Early Chalcolithic settlement (Isaiia, Romania). The archaeological campaigns of 2015 and 2017 offered remarkable results including a ceramic vessel, inside which an anthropomorphic figurine (with pregnancy depiction) and a small cone, both made out of clay, were found. Given the special character of the deposition, we collected several samples from the vessel and near it for phytolith analysis; samples of bone found next to the pot and from the nearby features were also dated by AMS radiocarbon. The palaeobotanical evidence based on the phytolith analysis showed that cereals and probably mugwort seem to have been in association with the small artefacts; both, and especially the latter, are known, aside from their practical uses (as aliment or remedy), as powerful symbols, used through the ages in magic practices. All of these facts nuance and augment the cultic interpretation of the deposition as a result of a ritual related to fertility (possibly to counteract some physiological problems or reproductive disorders) involving both feminine and masculine symbols and the use of plants.

**Abstract:**

The article presents the palaeobotanical investigations of a remarkable discovery from the Early Chalcolithic settlement of Isaiia–*Balta Popii* (Romania), a multi-layered site. The excavation of a dwelling brought to light a rather rare finding, meaning a medium sized ceramic vessel having deposited inside two objects of burnt clay: an anthropomorphic figurine depicting pregnancy attributes and a small cone. Given the special character of the deposition, several samples from the vessel and near it were collected for phytolith analysis. Our results highlighted a ritual plant deposition: Elongate dendritic and Blocky morphotypes suggest that cereals and probably *Artemisia* seem to have been used for this purpose. These plants are known, aside from their practical uses, as powerful symbols, used through the ages in magic practices. All of these facts are strong arguments to interpret this find as a result of a ritual related to fertility involving both feminine and masculine symbols and plant use.

## 1. Introduction

Following the intensification of farming and the population increase in the mid-Holocene, in the first half of the 5th millennia BC, the Early Chalcolithic Precucuteni–Trypillia A communities spread from the sub-Carpathian area up to the Prut, Dniester, and further to the Southern Buh Rivers (nowadays Romania, Republic of Moldova, and Ukraine), a vast territory where they transformed and evolved for the next hundreds of years. The good preservation of their settlements and clay artefacts has allowed archaeologists to interpret and reconstruct various aspects related to their material culture (architecture, ceramic technology) and spiritual life. The last fifty years of extensive research has brought to the attention of specialists a phenomenon that was linked to the spiritual life of these Early Chalcolithic people: the deposition of anthropomorphic figurines in ceramic containers, as attested through the discoveries from the settlements of present-day Eastern Romania: Isaiia–Iași County [1] and Poduri–Bacău County [2]. At each of the sites, one assemblage of 21 statuettes was discovered, deposited, along with miniature clay thrones and other small objects, in ceramic containers. While the statuettes at Poduri were found wrapped in “surprisingly well-conserved straws” [2] in a *matryoshka* system of vessels, the items at Isaiia were discovered in a clay container with soil in its interior [1]. From the way in which the soil accumulated in the vessel and from the deposition of the statuettes, it was clear that, in prehistory, they were also wrapped/put on something and not did lie on the base of the vessel. Unfortunately, at the time of the discovery (1998) no analysis was conducted on the sediment that accumulated in the vessel.

Recently, the latter site has provided another discovery of a deposition in a vessel [3], which gave us the occasion to perform phytolith analysis on the sediment from the vessel. Having a fairly long history, the study of phytoliths (microscopic opal silica particles that are produced in and between plant cells during the plant’s life) has been growing significantly for the last three decades, becoming an essential tool for palaeovegetation and paleoenvironmental reconstruction [4,5,6,7,8,9,10] as well as being a valuable proxy for paleoecology [11,12,13,14,15] and for the paleoclimate [16,17].

These ancient plant remains can also be a good tool for archaeological research [18,19,20,21], thus providing the opportunity to find data about the vegetal resources used by prehistoric communities [22,23]. Phytoliths have also been used successfully to establish the existence of agricultural practices [24,25] as well as to highlight aspects related to the diet of past communities [26,27] and past plant use [28,29].

In our study, we chose a different approach, that of using phytolith analysis in order to understand aspects related more to the spiritual life of the Early Chalcolithic communities. The analysis of phytoliths brings, implicitly, partial data about the plant resources (cultivated or from spontaneous flora) that the Precucutenian community from Isaiia–*Balta Popii* (north-eastern Romania) would have had at their disposal.

### 1.1. Isaiia–Balta Popii: Site and Context. Site Description

**Figure 1:**
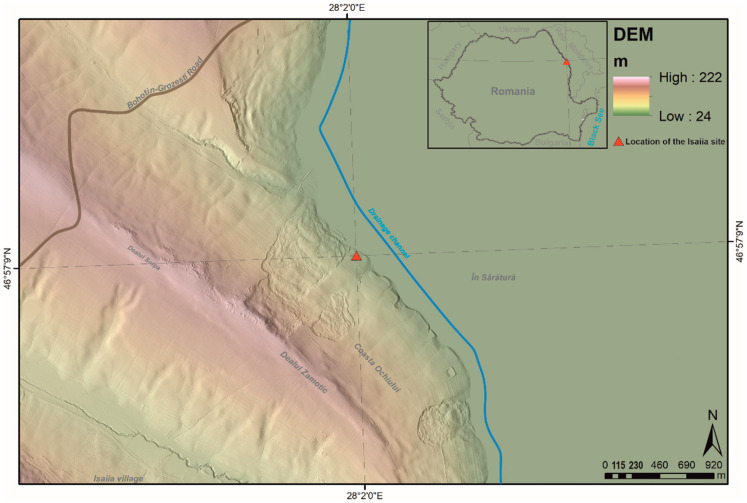
The location of the Isaiia site within Romania (black square) and within its local area; the site is marked by the red triangle on the hypsometric map. Source of the DEM: LiDAR data from the Romanian Water Administration, Prut-Bârlad branch, 1 × 1 m resolution (map by A. Asăndulesei).

**Figure 2:**
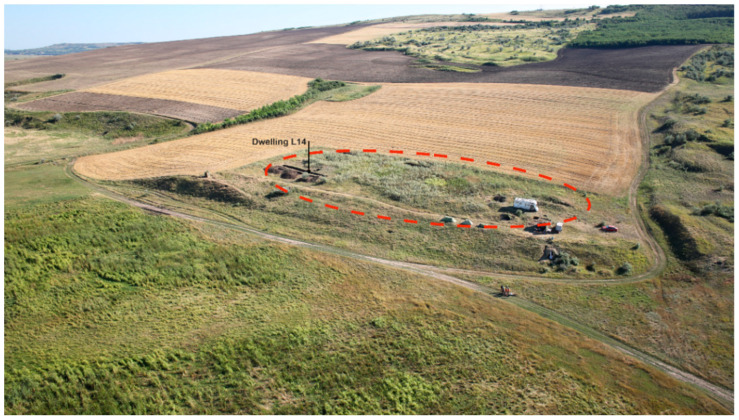
The aerial view (from the north) of the site’s landscape, with an indication (red dotted line) of its limits and the dwelling L14 (black arrow) (photo by A. Asăndulesei).

The archaeological site of Isaiia–*Balta Popii* (Răducăneni commune, Iași County, north-eastern Romania) is situated in the north of the Central Moldavian Plateau, in the Bârlad sub-unit (Long: 28°2′58.0″ E; Lat: 46°57′53.9″ N; Alt.: 30–35 m.a.s.l.) (Figure 1). It is located on the lower parts of the smooth slope of Zamotic Hill, being naturally delimited by two small torrential ravines (Figure 2). In this geographic area, the Jijia and Prut Rivers formed a common riverbed, ca. 4–5 km width, with temporary pounds characterised by a rich aquatic and riparian fauna and flora (for further details on the geography, see [1,30,31]). The area has always been favourable for habitation, being frequently attended by human communities throughout prehistory and history. Hence, the stratigraphy of the Isaiia–*Balta Popii* site is complex, with traces of human remains dating from the Neolithic (the *Notenkopf* phase of the LBK culture), Early Chalcolithic (Precucuteni Culture), Early Bronze Age, Early Iron Age (Hallstatt), 1st millennium AD (Sarmatic necropolis), and modern times [1]. The most consistent occupation of this place occurred during the Early Chalcolithic, when a settlement was founded that lasted for ca. 200 years, with an early, sporadic presence, followed by two main sequences of habitation.

**Figure 3:**
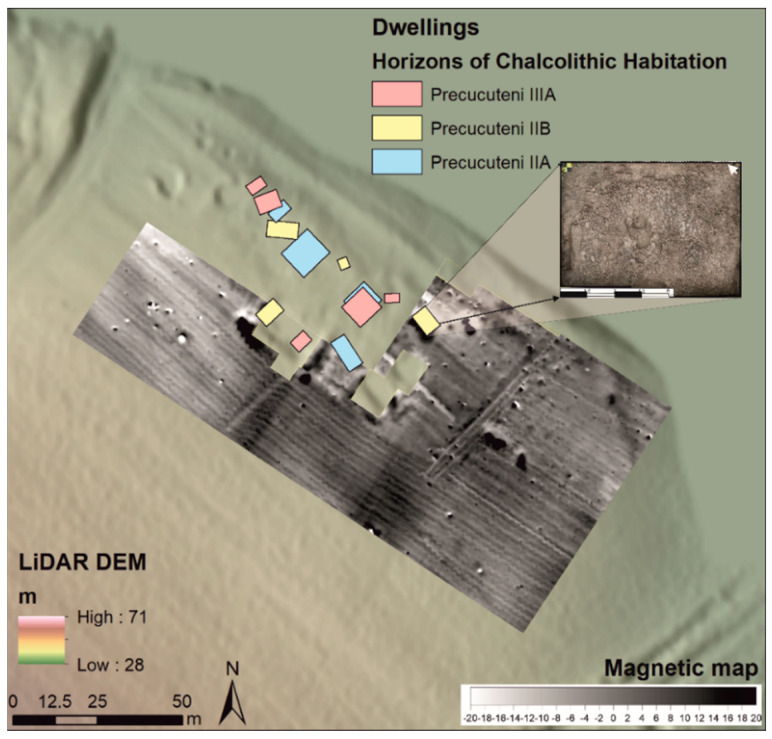
The magnetic map of the Isaiia site overlapped on the LiDAR DEM, with an indication of the Chalcolithic dwellings investigated thus far; the detail represents a vertical photography of dwelling L14 (map by A. Asăndulesei).

Due to 15 years of archaeological excavation (over several years from 1996 to 2020) and the geomagnetic survey of the site [31], it is known that the Early Chalcolithic settlement was of small dimensions (typical for its time–not exceeding 1 ha), with 4–5 surface dwellings in each sequence (Figure 3). Along with the dwellings, more than 50 pits and three stone structures were investigated; the latter, peripheral to the settlement, probably had a role in its symbolic delimitation [3]. For the Chalcolithic habitation, in recent years, a series of radiocarbon dating made on bone fragments from undisturbed contexts has been acquired [32].

### 1.2. Field Work (2015/2017): Dwelling L14-Setting and Findings

**Figure 4:**
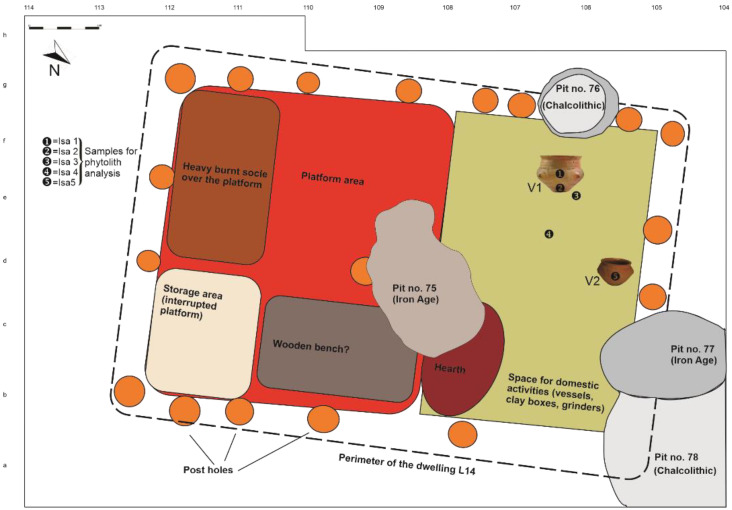
A schematic reconstruction of the floor of dwelling L14, with an indication of its main features and the samples for phytolith analysis.

Of interest for this paper was dwelling L14, excavated in 2015 and 2017, belonging to the IIB horizon of the Chalcolithic layer [3,32]. This dwelling, with the long axis orientated WNW–ESE, consisted in one room of medium dimensions (7.2 × 5.1 m); this was built in the wattle-and-daub tradition, with posts sustaining the walls and had a partial platform made of wooden beams, covered with a thick layer of clay (Figure 4) [3].

**Figure 5:**
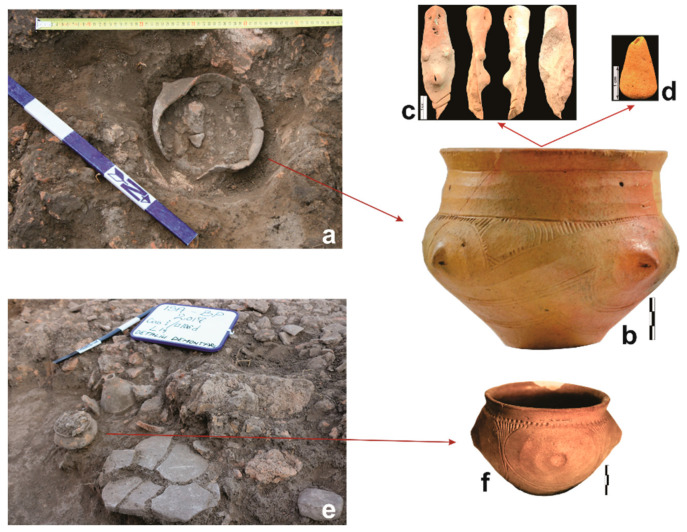
Artefacts discovered in the domestic area of the dwelling L14: (**a**) V1, in-situ; (**b)** V1, restored; (**c**) anthropomorphic figurine, found inside V1; (**d**) cone, found inside V1; (**e**) V2, in-situ; (**f**) V2, restored (photos by F.A. Tencariu).

In the presumed domestic area of the dwelling (without platform), under the heavy burnt remains of the walls, several intact or broken vessels were found. Between them, our attention was drawn by a decorated vessel (V1), typical for the second and third phase of the Precucuteni culture, which survived almost intact to the collapse of the wall (Figure 5a). Consequently, it was taken together with the soil within for sampling. The biconical vessel (13.5 cm height, 16.5 mouth diameter) has a symmetrical and very carefully made decoration realised by incisions and horizontal and oblique grooves; on the maximum diameter, it has four conical protuberances, each with horizontal unperforated holes (Figure 5b). At the time it was broken open in the laboratory, two objects of burnt clay were discovered inside: a fragment of an anthropomorphic figurine and a small cone (Figure 5c,d). These were not sitting on the bottom, but in the soil filling, close to the bottom, which means that they were placed on and probably covered with something, most likely an organic material. Only one metre and a half to the northeast, in the same area, two other small, intact vessels near a broken quern-stone were found (Figure 5e). One of the ceramic objects is a rather rare type of artefact, in the shape of a funnel with small holes all around its body–the so-called “smoke vessel” type. The other is a small (9 cm high), quasi-biconical goblet (V2), decorated with incisions, circular concentric grooves, and small round impressions; on its maximum diameter, it also has four chamfered protuberances (Figure 5f). The in situ position of these findings, together with the special content of the first, were sufficient reasons for an in-depth analysis: samples were collected from the area for radiocarbon dating as well as soil sampling from and around the vessels (V1 and V2) for the phytolith analyses.

## 2. Materials and Methods

### 2.1. Radiocarbon Dating

In order to establish the absolute chronology of the Chalcolithic habitation from Isaiia, especially dwelling L14 and other features near it (from the same layer), several samples (animal bone remains) were taken and sent for absolute dating (AMS 14C) at the Poznan Radiocarbon Laboratory (Poznan, Poland) and Beta Analytic (Miami, FL, USA). These were as follows: one sample from dwelling 14, right next to the V1 vessel; three from the stone assemblages marking the limits of the settlements (from the same layer as the dwelling); and three from the features adjacent to the dwelling (three pits from the same layer).

**Figure 6:**
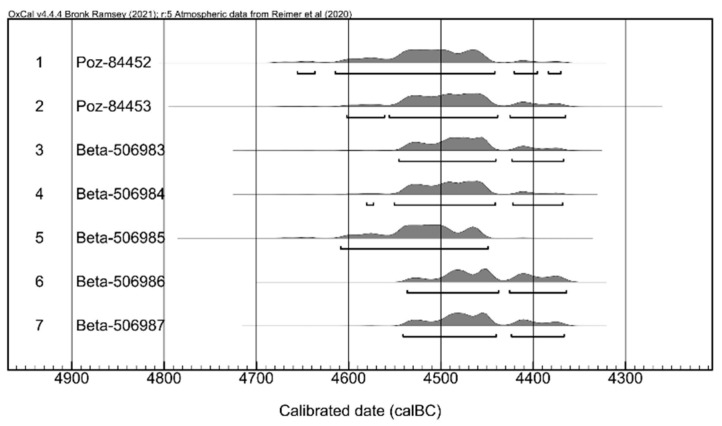
A multiple plot of the calibrated radiocarbon ages obtained from the samples from Isaiia [35,36].

The data obtained as a result of the AMS radiocarbon analysis of the seven samples were consistent with each other (Table 1, Figure 6), placing the habitation toward the middle of the 5th millennium BCE. These dates are in accordance with those revealed for other Middle Chalcolithic settlements from the area such as Poduri, Târgu Frumos, Târpești (Romania,) and Ruseștii Noi (Republic of Moldavia) and falls within the range proposed for the Precucuteni culture (ca. 4800–4350 BC) [33,34].

### 2.2. Phytolith Analysis (Sampling, Phytolith Extraction, Counting and Classification)

Out of several sediment samples collected for the phytoliths analysis, five were of special interest for this study: two samples from the V1 vessel, two reference samples (one from very close to V1 and the other from the floor of the dwelling, under the collapsed daub wall) as well as one sample from the V2 vessel (Table 2).

The phytolith extraction and chemical preparation were carried out at the Laboratory of Bioarchaeology at “Alexandru Ioan Cuza” University of Iasi, Iasi, Romania. Phytoliths were extracted from the sediment samples (1–3 g each sample) using a method adapted from techniques described by [37]: clay deflocculation with distilled water under magnetic stirring, 200 μm tumbling for the removal of coarse particles; centrifugation for 2000 t. min^−1^ for clay elimination; decarbonation with concentrated hydrochloric acid (33%) by heat and using an ultrasonic bath; organic matter oxidation under hot and ultrasonic action: KOH (10%), nitric acid (30%), hydrogen peroxide (30%); and phytolith densiometric separation with sodium polytungstate (density = 2.35). After cleaning, the residue was suspended in Zeiss immersion oil for mounting on glass slides. All of the prepared samples were analysed at the UMR 7264 CEPAM CNRS-Université Côte d’Azur (Nice, France). The slides were observed under a “Leica DMRB ™” microscope at 650× magnification (Leica Microsystems, Germany). Each phytolith was classified according to its morphology following the International Code for Phytolith Nomenclature 2.0 (International Committee on Phytolith Taxonomy [38], (2019). We identified and counted the phytoliths until we had counted at least 300 phytoliths (Table 2). As stated by Zurro [39], the standard count size, which varies from 250 to 300 phytoliths, should produce a precise and clear phytolith assemblage for archaeological studies. We mention that we did not notice any phytoliths that could not be included in a certain category according to the ICPT [38].

## 3. Results

### 3.1. Phytoliths—Identified Morphotypes

**Figure 7:**
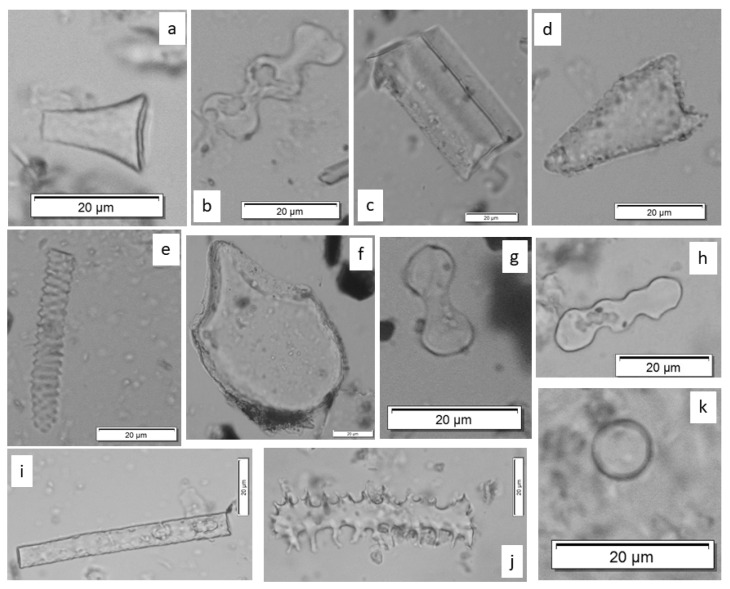
Examples of the phytolith morphotypes identified in the Isaiia samples: (**a**) Rondel; (**b**) Polylobate; (**c**) Blocky; (**d**) Acute bulbosus; (**e**) Tracheary; (**f**) Bulliform flabellate; (**g**) Bilobate; (**h**) Crenate; (**i**) Elongate entire; (**j**) Elongate dendritic; (**k**) Spheroid.

**Figure 8:**
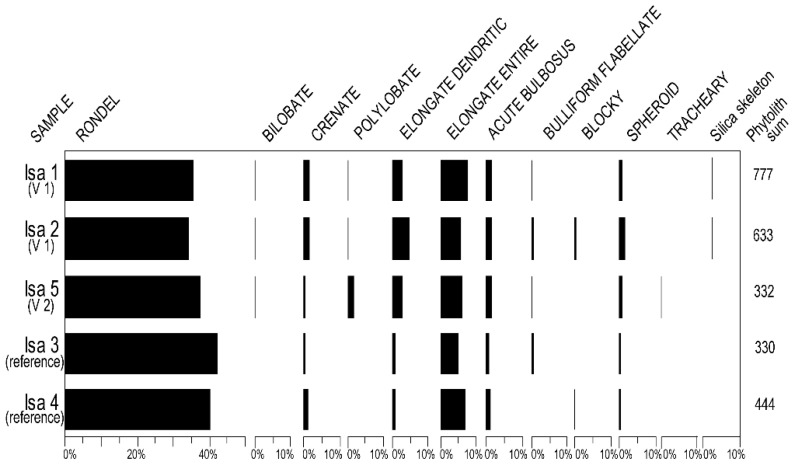
The phytolith diagram from the Isaiia site.

In this study, 11 morphotypes were distinguished: Rondel, Bilobate, Crenate, Polylobate, Elongate entire, Elongate dendritic, Acute bulbosus, Spheroid, Tracheary, Bulliform flabellate, and Blocky (Table 3 and Table 4, Figure 7 and Figure 8). *Silica skeletons* (articulated phytoliths) were also identified (Table 3, Figure 8). Rondel, Bilobate, Crenate, and Polylobate phytoliths were produced in the epidermal short cells of gramineous plants and can be used to identify the main subfamilies of the Poaceae family [40,41].

The Rondel morphotype is often associated with the Pooideae subfamily [42], plants with metabolism in C3, which grows in temperate environments and to which most cereals belong. Other subfamilies such as Arundinoideae may also produce this morphotype [43].

We also identified Crenate, phytoliths that are usually associated with the Pooideae subfamily [5]. Off the gramineous plants, this is the subfamily best represented in the temperate zone including here, for example, cereal species such as *Triticum dicoccum* (wheat), *Hordeum vulgare* (barley), and *Avena* (oats).

The Bilobate type of phytoliths was also observed in some of the samples. This morphotype is more abundant, especially for the Panicoideae and Arundinoideae subfamilies [43]. Panicoideae taxa are adapted to a warmer climate, with metabolism often in C4 and generally of intertropical distribution, with the exception of a few taxa (e.g., wild millet (*Setaria* sp.) or cultivated millet (*Panicum* sp.)).

The Elongate entire, Elongate dendritic, Acute bulbosus, and Bulliform flabellate phytoliths are mainly formed in the epidermis of gramineous plants [22,40,41,44]. However, these morphotypes can also be produced by other groups of plants [22,45].

The Blocky morphotype was identified in only a few samples, with a modest presence. This type has been assigned to the genus *Artemisia* [14,46,47,48], but also to some taxa of the Pinaceae family [14,38,46,49] and other gymnosperms (e.g., Cupressaceae, Taxaceae) [38,49]. This morphotype may also derive from the leaves of Cyperaceae and Poaceae [38].

Bulliform flabellate type phytoliths are located in the leaves, along the ribs, and allow the leaves to bend to avoid excessive water loss [50].

Elongate dendritic forms are produced at the level of inflorescences (glumes, lemma, palea) of Poaceae [51].

*Silica skeletons*, phytoliths that are connected [28], were identified only in the samples from the V1 vessel. These articulated phytoliths can either come from the vegetative parts of grasses (stems, leaves) or from their inflorescences, rarely preserved in a natural context. In the case of the present samples, it was a fragment of silicified epidermis from the grass inflorescence.

Spherical morphotypes (Spheroid) were also recorded. These forms are considered to be characteristic of dicotyledons [52,53,54,55,56]. A more accurate assignment cannot be made. Given the low phytolith production of dicotyledons compared to monocotyledons, relatively modest percentages of these morphotypes can be interpreted as significant.

The Tracheary morphotype was identified only once.

All of the identified morphotypes in the samples taken from Isaiia with their taxonomic attribution are presented in Table 4.

### 3.2. Phytolith Assemblages

In the Isa 1 sample, taken from the VI vessel discovered in dwelling L14 (more precisely from its base), we identified 777 phytoliths that we grouped into nine morphotypes, as follows: Rondel, Bulliform flabellate, Bilobate, Spheroid, Acute bulbosus, Elongate entire, Elongate dendritic, Polylobate, Crenate. *Silica skeletons* could also be identified (Figure 8). Rondel type phytoliths (over 70%) prevailed, followed by the Elongate entire (over 15%). Elongate dendritic phytoliths (over 5%) were also identified. The spectrum of phytoliths was supplemented by modest percentages of other morphotypes: Crenate (a little over 3%), and Spheroid (1.42%). The percentage of Spheroid phytoliths was not high (below 2%), but it should not be neglected at all due to the low phytolith production of dicotyledons. Acute bulbosus S phytoliths had a percentage of 2.57%. We also identified the Bilobate morphotype, but with a modest percentage (0.51%). Its presence in this context can provide very useful information for interpretation, especially if we consider the presence of Elongate dendritic phytoliths as well as silica skeletons.

Sample Isa 2, taken from the middle of the fill of V1, mostly highlighted the same morphotypes, predominant being the Rondel (68.24%) and Elongate entire (11.37%) types (Figure 8). Nevertheless, there were some obvious differences. Elongate dendritic phytoliths had almost double the percentage (9.16%) compared to the sample taken from the base of the vessel (5.41%). Additionally, in the case of the Spheroid type phytoliths, the percentage exceeded 3%, being more than 2-times higher than the one at the base of the vessel. Another difference is represented by the presence of Blocky type phytoliths (0.47%). Bilobate, Crenate, and Acute bulbosus phytoliths were also present, their percentages being close to the ones registered at the bottom of the vessel.

The analysis of the phytoliths extracted from the first reference sample (Isa 3), taken from the immediate vicinity of V1, highlighted the differences compared to the spectra obtained from the two samples in the vessel. The diversity of phytoliths was lower in this sample, with only seven morphotypes being recorded (Figure 8). Rondel phytoliths were also dominant in this sample, but the percentage was slightly higher (84.24%). The Elongate dendritic type was less than 2%, which showed an obvious decrease in this morphotype. There was also a decrease in the percentage of Spheroid type phytoliths: in this sample, they were less represented (less than 1%) than in the ceramic recipient. Bilobate, Polylobate, Blocky, Acute bulbosus, and *silica skeletons* phytoliths present in the vessel were not identified in this sample. The second reference sample (Isa 4), taken from under the adobe of the dwelling where the vessel was discovered, showed more resemblance to sample Isa 3. The spectrum of phytoliths (Figure 8) obtained for this sample also showed seven morphotypes: Rondel type phytoliths prevailed (80.41%), and Elongate dendritic recorded a low percentage (1.80%). The Spheroid morphotype had, as in the case of Isa 3 sample, a small percentage (0.45%).

The analysis of sample Isa 5, taken from the V2 vessel revealed 10 phytolith morphotypes (Figure 8): Rondel, Bulliform flabellate, Elongate entire, Elongate dendritic, Polylobate, Acute bulbosus, Spheroid, Tracheary, Bilobate, and Crenate. Rondel type phytoliths clearly prevailed (over 74%). These were followed by the Elongate entire morphotype at 11.75%. The Elongate dendritic type was present in 5.42%. The other morphotypes had lower percentages: Polylobate (3.61%), Acute bulbosus–2.41%, Spheroid–1.20%. All of the other identified morphotypes each showed 0.3%.

The main differences between the two types of samples (vessels versus references) are presented in Table 5.

## 4. Discussion

Usually, phytolith analysis is widely used to identify past plant taxa, providing data on the human use of plants (diet, agricultural practices, textiles, construction materials) or on extra-site palaeovegetation reconstruction. In this study, we had the opportunity to use these micro-palaeobotanical indicators to extract more information from a ritualistic context.

The circumstances and contents of the vessel found on the floor of the dwelling (V1) are clues indicating a ritualistic context, augmented and nuanced by the results of the phytolith analysis.

Starting with the in situ vessel itself, it may be relevant to specify that it was placed with the four protuberances oriented according to the cardinal points. Additionally, each protuberance has horizontal unperforated holes, and hence have no role in carrying or suspending the vessel; instead, they have the appearance of a stylised human face (very similar to the established rendering of the human face in the Vinça culture), possibly with an apotropaic role.

The figurine is rather rare within the anthropomorphic assemblage of the area, through the very suggestive display of pregnancy (Figure 5c). According to [57], only 2% of the statuettes of the Precucuteni communities depicted pregnancy attributes, achieved in two ways [58]. The first was by shaping the abdomen as swollen, as is the case of the statuettes from Isaiia, Târpeşti [59], Bosanci [60], Poduri [2], Româneşti [61], Costești [62], Ruseştii Noi I, Alexandrovka, and Holercani [63]. The second approach involved modelling the figurines with cavities in the abdomen and inserting small balls of clay (Ruseștii Noii: [63]). Both manners of modelling continued to be practiced in the Middle and Late Chalcolithic (see [58,64,65,66]). Child-bearing figurines were not restricted to the space of Precucuteni–Trypillia A culture, being present also in neighbouring contemporary cultures: Foeni (at Alba Iulia–*Lumea Nouă*, dated 4650–4450 cal BC: [67]), Turdaș (at Șoimuș–*La Avicola Ferma 2*: [68,69]), Gumelnița–Kodjadermen–Karanovo VI [70], and Hamangia [57].

The small clay cone has a hole at the top (Figure 5d), suggesting that in the past it was probably part of a two-part figurine, with a clay ball on top, joined by a wooden rod, similar to the pieces of the large cult assemblage discovered in dwelling no. 1 from Isaiia; it is very likely a stylized phalloid image—a masculine acolyte of the feminine figurine [1].

Our results show that phytoliths derived from grasses (Poaceae) dominated all of the spectra. Among this group, Pooideae is the best recorded subfamily. Our data also revealed the presence of herbaceous dicots and woody dicots. Beyond the specific information on the categories of plants at the site, our study highlights spiritual aspects. In support of our hypothesis are the percentages (5.41% and 9.16%) of the Elongate dendritic type phytoliths that were recorded in the two samples from the recipient with the statuette (samples Isa 1 and Isa 2, respectively). Additionally, in the vessel without the figurine (sample Isa 5), the percentage of Elongate dendritic exceeded 5%. Regarding both reference samples (Isa 3 and Isa 4), this percentage was below 2%. In this context, we can assume that intentionally spontaneous and most likely cultivated grasses (cereal inflorescences) were placed in the vessel.

The use of plants/seeds in activities with ritualic purposes was not unfamiliar to the Chalcolithic communities Precucuteni and Cucuteni. Thus, there are many known discoveries of macro-remains belonging to the species *Triticum dicoccum, Hordeum vulgare, Secale cereale, Bromus* sp., *Rumex acetosella, Rumex crispus, Vicia sativa, Sambucus nigra*, and *Cornus mas* inside ritualic complexes such as those from the Cucuteni A2 and B1 phases from the settlement of Poduri [71]. Similar discoveries were made at the Cucuteni B level from Sărata Monteoru, with seeds determined as belonging to *Hordeum* sp. and *Prunus* [72,73]. Additionally, ceramic recipients with small-sized seeds of *Triticum aevistum, Triticum dicoccum*, and *Hordeum* sp. were identified at Poduri. Due to their dimensions, the authors of the discoveries considered that these were harvested before reaching maturity, precisely so that they could be used in rituals or cultic ceremonies [73]. Among the seeds used in special activities, we have to mention the discoveries of *Lithospermum officinale* seeds. These were identified, in high number, in the Cucuteni A2 levels from Poduri [71] and Izvoare [73], but also in Cucuteni B at Frumușica [74], the context of the discoveries suggesting their usage for creating necklaces or during magical/medicinal activities. The existence of a relation between the seeds belonging, especially to cereals, and the religious environment is also proven by the cult ensemble, discovered in the Precucuteni III level from Poduri. Inside a large pot, covered with a lid and protected by another vessel, placed upside down, 21 feminine statues, 13 thrones, and two other small-sized objects were identified. The figurines were kept, inside the recipient, in cereal straws, which is why there was a double significance: with the help of the straws, the figurines were protected, however, they also ensured the sacralisation of the effigies of the deities, precisely through contact with these plants [58]. Similar practices were attested in Tripolie A, with cereal grains being included in the paste of the female statuettes [75]. Last but not least, we have to mention the usage of cereal seeds, and not only during practices specific to the so-called “skull cult” attested to in the Cucuteni A2 level from Poduri [76]. Inside a pit, a skull was identified as belonging to a child, towards the centre of an oval-shaped lens consisting of soil mixed with various carbonised seeds, among which the following were determined: *Triticum dicoccum, Triticum aestivum, Triticum monococcum, Triticum dicoccoides, Hordeum vulgare, Secale cereale, Avena sativa, Chenopodium album, Rumex crispus, Polygonum convolvulus, Cerasus avium, Atriplex* sp., *Polygonum aviculare, Panicum miliaceum, Vicia* sp., *Rubus idaeus, Polygonum hydropiper, Thlaspi arvense*, and *Brasica nigra* [77].

Ethnographically, there are also countless accounts related to the symbolic utilisation of cereal seeds (especially wheat) in fertility related rituals (marriage, child-bearing, etc.) or apotropaic practices [78,79].

The share of Spheroid phytoliths most likely shows the presence of woody dicotyledons. Leaves or probably very young dicotyledonous stems/branches were used in the ritual of the Isaiia community. The Blocky phytoliths reinforce the idea of the presence of dicotyledons in the vessel with the statuette. Their origin could be *Artemisia* sp. (mugwort), the presence of conifers being less likely in this low area (30–35 m.a.s.l.). According to Blinnikov [47], Blocky phytoliths of *Artemisia* seem to be comparable to those of *Abies* and *Picea*, but as stated by An and Xie [49], the Blocky morphotype produced by Pinaceae usually has bordered pits on the surface. We did not notice pits on the surface of the Blocky phytoliths we observed. Moreover, the Blocky identified by us resembled the type attributed by Morris [48] to *Artemisia*. We believe that it could be *Artemisia* sp., given its many uses and benefits as an aromatic, medicinal, ornamental, and, not least, a ritual plant. Furthermore, we also considered the archaeological context (a ritualistic one), the composition of the phytolith assemblage of the V1 vessel, and the percentage recorded by Spheroid (which suggests the presence of dicotyledons).

There are testimonies in the mythological, ethnographic/ethno-botanic, and magic medicine literature of the medical and apotropaic use of several species of *Artemisia*. From Antiquity to modern times, these plants have formed part of the remedies employed in healing a large spectrum of health problems—“the mother of all herbs” [80,81]. When used for medicinal purposes, they were prepared in diverse ways as decoctions, fumigation, bruised, combined with wine, oils, honey, etc.

In the medical papyri of Ancient Egypt, in Assyrian texts [82,83], in the works of doctors (Dioscorides, Soranus) from Antiquity [84,85], in the scholarly and folkloric texts from Medieval and rural Europe [83,86,87,88,89,90], *Artemisia* sp. are indicated as ingredients of cures for treating various diseases. These ailments are related to the digestive system, wounds, epilepsy, lung diseases, headaches, malaria, kidney disorders, scrofula, tooth aches, sore throat, gingivitis; mugwort was also seen as a cardiac stimulant, anti-diabetic, antidote for some poisons, and as a repellent of the parasites that can populate the human body (both internal as worms, and external as flees and bedbugs) [82,83,84,85,86,87,88,89,90,91].

What is to be remarked is the fact that *Artemisia* sp. has had a strong feminine valence in the medical/pharmaceutical literature since the beginning of the first millennium A.D. According to Pliny the Elder, there were opinions according to which the name of the plant *Artemisia* is derived from Artemis Illythia [91] (25.36). Illythia/Eileithyiai was the goddess whose function was to assist women in parturition [92,93,94]. Pliny uses this name as an attribute of Artemis; in Delos, considered the mythical place where Artemis and Apollo were born, the Eileithyiai goddesses and Artemis were assimilated [93]. The same author also speaks of the fact that *Artemisia*, prepared as a pessary in combination with oils, is curative of diseases of the uterus, while its roots, taken in drink, are a powerful purgative that can help to expel the dead foetus [91] (26.90). As a decoction, it was used in baths or drunk for menstruation and after birth problems [91] (26.90).

Dioscorides wrote, also in the first century A.D., that *Artemisia absinthium* expels the menstrual flow [84] (5-49), that *Artemisia pontica* can induce the end of menstruation [84] (3–29), while *Artemisia monoklonos* is good to be put into women’s baths for driving out the menstrual flow and afterbirth [84] (3–127). He speaks of it as an abortifacient for the closure and inflammation of the womb [84] (3–127). Some decades later, the physician Soranus of Ephesus also provided information about the risk of abortion coming from *Artemisia* usage [85] (XV; XVII), but also indicated *Artemisia* as an ingredient of medicines for uterine pneumatises [85] (LVI), menstrual delays and pains [85] (XLVIII), and pica [85] (XV.52).

In the Middle Ages, the *Artemisia* plant was still employed as a treatment in female problems (see [83]); even nowadays, in traditional societies, beliefs in its benefactor role on women still exist. In the folkloric/ethnographic literature of Romania, *Artemisia* sp. are still indirectly associated with Artemis, being called “the herb of the virgins”, but only a few mentions on its role in gynaecologic affections have been made [89,95]. The apotropaic role of this plant considered as protecting people against the evil spirits that might drive them mad or handicap them is more significant [89,96,97,98,99]. The plant was also known for its hallucinatory effects, being used in some traditional rituals [100].

Unlike the frequency of other plants/seeds (especially cereals) found in non-domestic contexts, we are not aware of the existence of any other Chalcolithic deposition of *Artemisia* sp. Nevertheless, even at a glance at the medical and symbolic significance of *Artemisia* sp. over time, as highlighted above, there is no doubt that this widespread spontaneous plant was known and used in prehistory as a remedy as well as a magical herb.

## 5. Conclusions

At this point, there are enough premises to risk a reasonable interpretation of this find. First, we have an in situ vessel (sitting on the floor, towards the west corner of a dwelling), whose position and stylistic attributes already point to a possible ritualistic behaviour. Second, its tangible contents support and enhance the plausibility of this interpretation: the feminine statuette, presumably fragmented intentionally and displaying pregnancy attributes, accompanied by the small cone, part of a former masculine representation. Last but not least, the ‘invisible’ content of the vessel, revealed by phytolith analysis, which protected the ‘couple’ of clay objects, consisted of cereals and, probably, mugwort; both are plants with a notable symbolic load, aside from their economic, dietary, or medical importance. Therefore, we think that this vessel and its contents represent the paraphernalia of a ritual, probably intended to prevent or to cure fertility problems. The fact that the figurine is fragmented, and the cone lacks the small ball (the ‘head’) is not accidental; the deposition, in the vessel, of the clay objects, wrapped in a plant coat, most likely took place after the performance of a ritual, but without losing its magical significance. Moreover, this could also be an argument in favour of the magic and medicine man in one person (the shaman), which may look like a truism, but in fact is an axiom with little archaeological evidence behind it.

A ‘running joke’ tells that archaeologists name ‘ritual’ as everything they do not understand ‘functionally’. The truth behind this joke is that identifying and defining prehistoric ritual still undergo failures, primary because of our contemporary ‘need’ to separate the sacred from the profane, rooted in the post-Enlightenment rationalism [101]. The context of these finds as well as their tangible and microscopic content allowed us to catch a glimpse at the complexity and symbolism that links the attitudes, actions, experiences, and the materiality of prehistoric people. Ritual practices in prehistory, regardless of how vague or incomprehensible they may appear nowadays, were, without any doubt, omnipresent, and most probably profoundly intertwined with all other aspects of everyday life.

## Figures and Tables

**Table 1:** The radiocarbon data from the Chalcolithic settlement of Isaiia.

No.	Context Description	Lab. No.	^14^C Age (BP)	Material	Calibrated BC Range (68.2%)	Calibrated BC Range (95.4%)
1	Dwelling no. 14 (floor, domestic area)	Poz-84452	5680 ± 40	bone	4545 (68.2%) 4461	4669 (0.6%) 4660
						4654 (1.2%) 4639
						4618 (91.7%) 4446
						4420 (1.8%) 4400
2	Stone assemblage, eastern edge of the settlement	Poz-84453	5660 ± 40	bone	4537 (68.2%) 4456	4592 (85.3%) 4438
						4426 (10.1%) 4370
3	Stone assemblage, southern edge of the settlement	Beta-506983	5650 ± 30	bone	4517 (68.2%) 4455	4547 (88.1%) 4444
						4421 (5.8%) 4395
						4385 (1.6%) 4374
4	Stone assemblage, southern edge of the settlement	Beta-506984	5660 ± 30	bone	4520 (68.2%) 4459	4554 (92.1%) 4445
						4421 (2.9%) 4397
						4381 (0.4%) 4375
5	Pit no. 76	Beta-506985	5690 ± 30	bone	4548 (65%) 4486	4604 (95.4%) 4456
					4471 (3.2%) 4466	
6	Pit no. 78	Beta-506986	5630 ± 30	bone	4500 (55.7%) 4446	4531 (95.4%) 4369
					4419 (12.5%) 4400	
7	Pit no. 79	Beta-506987	5640 ± 30	bone	4519 (68.2%) 4449	4542 (79.1%) 4441
						4425 (16.3%) 4371

**Table 2:** The samples analysed regarding the phytoliths.

Sample Code	Context	Phytolith Sum
Isa 1	V1 vessel—the fill from its bottom	777
Isa 2	V1 vessel—the fill from the middle	633
Isa 3	Reference (near V1 vessel)	330
Isa 4	Reference (floor of the dwelling)	444
Isa 5	V2 vessel—the fill	332

**Table 3:** The phytolith data from Isaiia.

Sample Code	rondel	Bulliform flabellate	Spheroid	Acute bulbosus	Elongate entire	Elongate dendritic	Crenate	Polylobate	Bilobate	Blocky	*Silica skeletons*	Tracheary	Phytolith Sum
Isa 1	551	1	11	20	120	42	24	3	4	0	1	0	777
Isa 2	432	3	20	19	72	58	21	2	2	3	1	0	633
Isa 3	278	2	3	5	33	6	3	0	0	0	0	0	330
Isa 4	357	0	2	9	59	8	8	0	0	1	0	0	444
Isa 5	247	1	4	8	39	18	1	12	1	0	0	1	332

**Table 4:** The phytolith morphotypes identified at Isaiia with their taxonomic attribution and the corresponding references.

Morphotypes	Main Taxonomic Attribution	Bibliography
Elongate entire	Poaceae	[22,40,41,44]
Elongate dendritic	Poaceae	[22,40,41,44,51]
Acute bulbosus	Poaceae	[22,38,40,41,44]
Bulliform flabellate	Poaceae	[22,40,41,44]
Rondel	Poaceae, Pooideae	[40,41,42,43]
Crenate	Poaceae, Pooideae	[5,40,41]
Spheroid	cf. Dicotyledonous	[52,53,54,55,56]
Bilobate	Poaceae, Panicoideae/Arundinoideae	[40,41,43]
Polylobate	Poaceae, Panicoideae	[38,40,41]
Tracheary	cf. Dicotyledonous	[45]
Blocky	Poaceae, Pinaceae, Cupressaceae, Taxaceae, *Artemisia*	[14,38,46,47,48,49]

**Table 5:** The main differences between the phytolith assemblages of the vessels versus the reference samples.

Criteria	Vessels	Reference Samples
Elongate dendritic	Up to 9.16%	Up to 1.82%
Spheroid	Up to 3.15%	Up to 0.9%
*Silica skeleton*	Up to 0.15%	absent
Bilobate	Up to 0.51%	absent
Polylobate	Up to 3.61%	absent
Tracheary	0.3%	absent
Phytolith diversity (number of morphotypes)	11	8

## Data Availability

All data are included in the manuscript.
